# Location-based collective distress using large-scale biosignals in real life for walkable built environments

**DOI:** 10.1038/s41598-023-33132-z

**Published:** 2023-04-12

**Authors:** Jinwoo Kim, Ehsanul Haque Nirjhar, Hanwool Lee, Theodora Chaspari, Chanam Lee, Youngjib Ham, Jane Futrell Winslow, Changbum R. Ahn

**Affiliations:** 1 Department of Architectural Engineering, Gachon University, 1342, Seongnam-daero, Sujeong-gu, Seongnam-si, Gyeonggi-do 13120 South Korea; 2 Department of Computer Science & Engineering, Texas A&M University, 330 Peterson Building, 435 Nagle St, College Station, TX 77843 USA; 3 Department of Landscape Architecture and Urban Planning, Texas A&M University, Scoate Hall 106, 3137 TAMU, College Station, TX 77843 USA; 4 Department of Computer Science & Engineering, Texas A&M University, 329 Peterson Building, 435 Nagle St, College Station, TX 77843 USA; 5 Department of Landscape Architecture and Urban Planning, Texas A&M University, Scoates Hall 107C, 3137 TAMU, College Station, TX 77843 USA; 6 Department of Construction Science, Texas A&M University, 329B Francis Hall, 3137 TAMU, College Station, TX 77843-3137 USA; 7 Department of Landscape Architecture & Urban Planning, Texas A&M University, A332 Langford Architecture Building, 3137 TAMU, College Station, TX 77840 USA; 8 Department of Architecture and Architectural Engineering, Seoul National University, 39-404, 1 Gwanak-ro, Gwanak-gu, Seoul, 08826 South Korea

**Keywords:** Civil engineering, Quality of life

## Abstract

Biosignals from wearable sensors have shown great potential for capturing environmental distress that pedestrians experience from negative stimuli (e.g., abandoned houses, poorly maintained sidewalks, graffiti, and so forth). This physiological monitoring approach in an ambulatory setting can mitigate the subjectivity and reliability concerns of traditional self-reported surveys and field audits. However, to date, most prior work has been conducted in a controlled setting and there has been little investigation into utilizing biosignals captured in real-life settings. This research examines the usability of biosignals (electrodermal activity, gait patterns, and heart rate) acquired from real-life settings to capture the environmental distress experienced by pedestrians. We collected and analyzed geocoded biosignals and self-reported stimuli information in real-life settings. Data was analyzed using spatial methods with statistical and machine learning models. Results show that the machine learning algorithm predicted location-based collective distress of pedestrians with 80% accuracy, showing statistical associations between biosignals and the self-reported stimuli. This method is expected to advance our ability to sense and react to not only built environmental issues but also urban dynamics and emergent events, which together will open valuable new opportunities to integrate human biological and physiological data streams into future built environments and/or walkability assessment applications.

## Introduction

Built environments, from neighborhoods to cities, have been connected to various physical and mental health outcomes and risk factors such as hypertension, respiratory disease, asthma, and obesity^1–3^. Both prolonged and short-term exposure to negative environmental stimuli (e.g., litter, partially demolished houses, graffiti, abandoned vehicles, unattended dogs, and so forth) is shown to trigger physiological stress symptomatology that increases the risk of chronic illness^1^. Moreover, negative environmental stimuli can limit the mobility, especially walking, of disadvantaged and vulnerable populations (e.g., children, older adults, and people with mobility, cognitive, vision, and hearing impairments)^4^. Thus, evaluating the built environment and its impact on urban citizens’ health and wellbeing is an important research and policy priority.

Recently, the increasing use of wearable sensors and location-aware mobile devices has created unprecedented opportunities to assess urban built environments by capturing citizens’ distress levels, beyond the inherent limitations (e.g., subjectivity concerns, and time and labor intensiveness) of the conventional approach^5,6^. Although directly measured field audit data (e.g., presence of litter, excessive noise, and poor air quality) and passively collected urban context data (e.g., user-generated data, volunteered data, and infrastructure-related data) are powerful traditional measures, those measures do not provide the real-time momentary experiences of the environment by urban citizens^7^. To mitigate the limitations of traditional methods, several interdisciplinary efforts have shed light on the potential to capture and assess the human response to urban built environments in an unobtrusive and continuous way using biosignals, including electrodermal activity (EDA)^8–10^, gait patterns^11,12^, heart rate^13,14^, and brain activity^15,16^.

Pedestrians’ biosignals contain unique information regarding their interactions with the built environment that offer valuable insight into the role of urban spaces^17–19^. Accordingly, biosignals have been used to assess the degree of distress felt by pedestrians associated with external stimuli while walking^20–22^. Data can be analyzed with regard to gender^7^, age^23^, and degree of disability^24^, offering more population-specific and objective evidence than existing techniques. EDA and heart rate from photoplethysmography or electrocardiography have also been examined to portray the psychophysiological state associated with emotional arousal toward stressors^25–29^. Gait pattern is considered an effective measure for understanding pedestrian responses to the physical attributes of the environment such as curb ramps, slope conditions, and sidewalk defects^30–32^. Specifically, the effectiveness of gait patterns in capturing the impact of negative environmental stimuli has been studied. For example, the feasibility of using gait data (e.g., stride length and gait speed) was investigated to evaluate the impact of environmental demand for mobility^33^. Their results showed considerable variability in gait responses in high-demand environments (e.g., sidewalk defects and lack of traffic signals) in naturalistic ambulatory settings^33^.

Despite the importance of understanding environmental contributors to pedestrian distress, most prior research on this topic has been conducted in virtual or controlled environments^32^. Everyday environments where people live, work, play, and learn are dynamic and subject to various external factors. Therefore, its applicability to real-life settings is limited. Current understanding is lacking as to whether biosignals from real-world settings can capture pedestrians’ distress responses effectively. Specifically, capturing the distress of pedestrians in real life poses various technological and implementation challenges such as (1) signal noise from confounding factors in real-life environments (e.g., motion artifacts, improper sensor placement, and weather conditions)^33^; (2) difficulties in obtaining biosignals and locational data together with trip mode information (e.g., lack of platform for data upload and acquisition during the daily life of each subject, and distinguishing walking trips from other outdoor activities such as running, bicycling, driving, etc.)^27^; (3) lack of experimental protocols in defining and collecting the ground truth in the distress assessment of subjects during their daily lives^13,34^; and (4) inherent challenges with big data (e.g., a massive amount of data from different sensors, algorithm complexity, and various parameters/variables such as segment lengths). Therefore, a more thorough investigation is essential to answer the question of whether biosignals have the potential to capture the environmental distress of pedestrians in their daily lives.

In this context, this study (1) examines the extent to which different types of negative environmental stimuli (e.g., poor walking surfaces, blocked sidewalks, unattended dogs, etc.) are associated with changes in biosignals (i.e., EDA, gait patterns, and heart rate) in real-life settings; and (2) investigates a machine learning model for spatial biosignals to capture location-based collective distress. In particular, the effects of segment granularity and interplay of different biosignals are investigated. The paper is structured as follows. First, we introduce newly collected location-based large-scale biosignals that cover 191.52 km of walking paths from 67 subjects over 5 months. Next, we present ways to preprocess biosignals. The preprocessed data are analyzed via statistical and machine learning models to identify significant negative environmental stimuli at the individual level, as well as at the collective level aggregating all individual-level data. Results from this study will provide new insights into built environmental assessment, in which computational systems collaborate with the psychophysiological data to jointly identify spatiotemporal moments of interest in our urban built environments.

## Method

### Data collection

Field experiments were performed in Bryan and College Station, Texas, from February 10, 2021 to June 28, 2021. The data collection protocol was approved by the Texas A&M University Institutional Review Board (IRB2019-0059D). Informed consent was obtained from all subjects. All method were carried out in in accordance with relevant guidelines and regulations. A total of 67 subjects between 18 and 78 years of age (M = 31.52 and SD = 17.33) were recruited using the University’s bulk email system and from the flyers distributed to community associations in the study area. Additional eligibility criteria included being able to walk for about 30 min without another person’s assistance, and not having a disability or condition that makes it difficult for outdoor walking, such as mobility, vision, hearing, or cognitive impairments. Older adults (aged 65 or older) were requested to complete a pre-screening survey to further confirm their eligibility as they are more likely to have conditions that make their participation in this study difficult or inappropriate. The telephone-based Mini-Mental State Examination tool and mobility, vision, and hearing assessment questionnaires were used as the pre-screening instrument.

**Figure 1:**
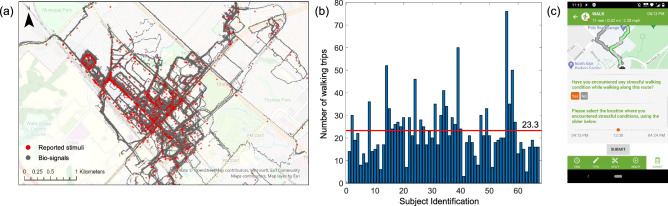
Data collection overview: (**a**) spatial distribution of biosignals and reported stimuli from subjects; (**b**) number of walking trips for each subject; and (**c**) screenshot of the Daynamica application. The image in (**a**) is created using the ArcGIS Pro 3.0, https://www.esri.com/en-us/arcgis/products/arcgis-pro/overview. The image in (**c**) is created using the Daynamica, https://daynamica.com/.

Each subject was asked to conduct the data collection while going about their outdoor activities during a 2-week long period of daily life while wearing two sensors [a wristband (Empatica E4^35^)] on their nondominant hand and a smartphone worn on their waist using a provided fanny pack). Participants were asked to delay the data collection or skip the days with inclement weather conditions or otherwise perceived to be unsafe or undesirable (e.g., rain, heat, wind, tornado, hurricane). The wristband collected the EDA (4 Hz) and blood volume pulse (BVP) (64 Hz) data. The smartphone collected accelerations, angular rates, and magnetic field data (50 Hz) from an inertial measurement unit (IMU) and Global Positioning System (GPS) data (1 point per 3 s). During the experiment, participants were requested to complete a survey using a smartphone-based application (customized Daynamica application^36^) for each walking trip to report the trip/activity mode and purpose(s) and the locations/types of associated environmental stimuli. The smartphone application automatically detects outdoor trip modes (e.g., driving, walking, biking, etc.) and provides a survey immediately after the completion of a walking trip. The specific route participants walked for each completed trip was displayed on a map and, using a time slider bar (see Fig. 1c), they were asked to report the location where they experienced distress and the specific types of environmental features/stimuli that triggered their stress response. The wristband was connected to the smartphone application and all data collected were automatically uploaded to the cloud server on a daily basis. This allowed us to monitor the participants’ data for daily quality checks. To account for the potential bias or unusual patterns in the data as participants learn about the experimental protocol, we discarded the first one-day data from the final analysis. The 14-day data starting from the second day of the experiment, therefore, was used for the study.

**Figure 2:**
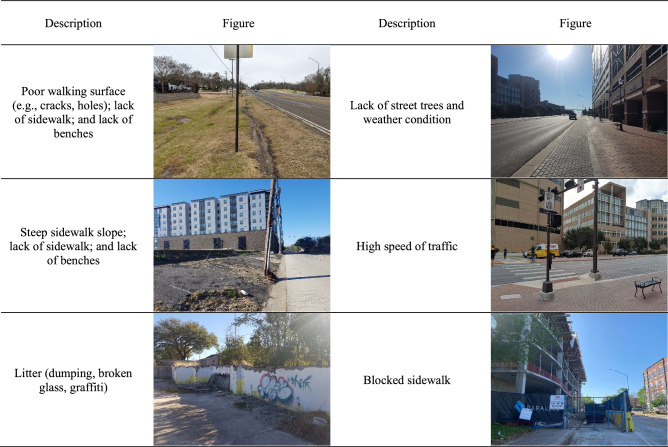
Examples of negative environmental stimuli in the study area.

As shown in Fig. 1a, the spatial distribution of the collected data covers mixed-use residential neighborhoods (Northgate, Boyett, Hyde Park, Oak Terrace, Woodson Acres, College Hills Estates, and College Hills Woodlands) as well as the Texas A&M University campuses in Bryan and College Station, Texas. This area contains diverse residential, recreational, educational, and commercial land uses including elementary schools, a large public university, retail stores, restaurants, bars, cafés, pharmacies, banks, restaurants, gyms, parks, and so forth. The total length of walking paths by all subjects in this study was 191.52 km. The collected data sets included geocoded biosignals (EDA, BVP, and IMU with GPS) from a total of 1,561 walking trips from 67 subjects and 1,581 locations of stimuli and experienced stress levels during their walking trips. The number of walking trips by each subject is presented in Fig. 1b and ranged from 3 to 76 trips per 14 days, with an average of 23.3 trips. The types of reported stimuli included both the presence of undesirable conditions/elements (9 types) and the absence of desirable conditions/elements (5 types): (1) poor walking surface (e.g., cracks, holes), (2) blocked sidewalk, (3) steep sidewalk slope, (4) litter (dumping, broken glass, graffiti), (5) abandoned vehicle, (6) high speed of traffic, (7) unattended dogs, (8) rowdy people, (9) weather condition, (10) lack of sidewalk, (11) lack of crosswalk, (12) lack of benches, (13) lack of crosswalk and pedestrian light, (14) lack of street trees. In some locations only one of these conditions exited where in some other locations, multiple of these conditions coexisted. Figure 2 presents sample images of negative environmental stimuli in the study area.

### Data preprocessing and feature extraction

This research used the EDA, IMU, and BVP signals to analyze the impact of negative environmental stimuli in real life. The raw EDA signal was preprocessed using a Bateman low-pass filter of 12-sample lengths to smooth the EDA^36–38^. We then screened out unusable EDA data based on the following criteria: (1) mean EDA below 0.01 µS; (2) no skin conductance response (SCR) frequency; and (3) SCR frequency over 25 counts. Additionally, a visual inspection was conducted to confirm missing signals and/or high levels of noise^37–40^. As a result, a total of 1,325 walking trips with valid/usable EDA were selected for this study.

To preprocess raw IMU data, the Butterworth low-pass filter with a cutoff frequency of 4 Hz was used to reduce high-frequency noise in IMU data^41,42^. Next, filtered IMU was examined to identify intervals/interruptions (nonwalking or unusable IMU) in the walking trip using the following four steps: (1) magnitude was computed to convert the three directions of acceleration; (2) a short-time Fourier transform was used with a 3 s time window; (3) intervals were determined and the corresponding data were discarded if a segment was below 0.5 amplitudes of the spectral components for 15 s; and (4) manual inspection was performed to confirm the identified intervals^42^.

To calculate heart rate from BVP data, the Butterworth band-pass filter was used for noise removal (0.4–0.6 Hz and 4–8 Hz cutoff). Then, the discrete wavelet transformation method was applied for motion artifact removal. Heart rate was calculated from the raw BVP signal using the Biosignal Processing in Python library^43^. The final heart rate measure was refined using a moving average filter, leading to a total of 1310 walking trips with BVP.

Raw features were extracted from each biosignal. Skin conductance level (SCL), SCR amplitude, and frequency were extracted from EDA^37,43^. Stride time as a representative feature of gait pattern was computed from the IMU data^20^. Mean heart rate was calculated from the BVP data. We also employed a saliency detection approach to extract additional meaningful features from the biosignals. This method allows to capture distinctive physiological responses by calculating the contrast between the current signal segment and its neighbors^11,32,41,43^. The analogous signal portion of the biosignals is firstly segmented using a non-fixed-length approach (bottom-up segmentation). A representative attribute for each biosignal was computed after determining segment boundaries. Following that, physiological saliency cue (PSC) values were calculated by comparing one segment's distinctiveness to other segments. A PSC can be discretely operationalized for each of the representative attributes calculated from biosignals. The PSC values for gait patterns and BVP are denoted as IMU PSC and BVP PSC, respectively. PSC values for EDA are denoted as EDA PSC.

### Association between biosignals and self-reported negative environmental stimuli

**Figure 3:**
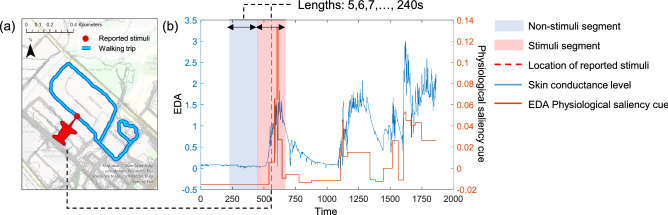
Nonstimuli and stimuli segments of the reported environmental stimulus from a walking trip of a subject: (**a**) visualization of a walking trip on the app-based map; and (**b**) graph of biosignals and negative environmental stimulus location image in (**a**) is created using the ArcGIS Pro 3.0, https://www.esri.com/en-us/arcgis/products/arcgis-pro/overview.

Biosignals were collected for each walking trip from each subject (see Fig. 1b). We first examined short-term physiological reactivity to multiple negative environmental stimuli using the biosignals collected in real life. Figure 3a and b indicates a sample graph and walking trajectory for one subject’s biosignals from one walking trip and the negative environmental stimuli the subject experienced. The location of each negative environmental stimulus is represented by a red vertical dotted line, which was determined based on the smartphone survey reports completed by the subject. The blue and red shaded sections in Fig. 3b present a window of the nonstimuli segment and stimuli segment, respectively, from the stimulus location point reported by the subject. Previous research confirmed that apparent physiological reactivity patterns are shown 5–10 s after passing the target stimuli in a controlled setting^9^. Thus, we tested the window size starting from 5 s and by increasing in 1-s increments. The window size ranged from 5 to 240 s in each nonstimuli and stimuli segment was tested. The SCL and EDA PSC showed an immediate change in amplitude/frequency responding to the negative environmental stimuli [e.g., blocked sidewalks and litter (dumping, broken glass, weeds, and graffiti)].

A linear mixed-effects (LME) model was used to examine the impact of the negative environmental stimuli on the biosignals of pedestrians. Specifically, the LME model was used due to its ability to allow the modeling of both fixed and random effects. Mixed effects models are useful when we have data with more than one source of random variability. The outcome of this study was measured more than once on the same participant (3–76 trips per participant). The data accounts for both within-person and across-person variability. A single measure of residual variance can’t account for both. Weather effects in this short time period (window lengths from 5 to 240 s for each nonstimuli segment and stimuli segment) might not be significant on each walking trip. However, biosignal change patterns among participants might be different due to weather changes because each participant repeatedly measured the biosignals for 2 weeks in different weather conditions. Thus, LME model was used due to its ability to allow the modeling of both fixed and random effects^44^. The subject identification was used for random effects. The LME equation is defined as follows:

1 $${\text{y}}_{{\left\{ {{\text{ij}}} \right\}}} = {\upmu } + {\upbeta } \times {\text{x}}_{{\left\{ {{\text{ij}}} \right\}}} + {\text{u}}_{{\text{i}}} + {\text{e}}_{{\left\{ {{\text{ij}}} \right\}}}$$

where $y_{{\left\{ {ij} \right\}}}$ = response variable (biosignals) for participant *i* over environmental stimuli $j$; and $x_{{\left\{ {ij} \right\}}}$ = reported stimuli information by participant *i* regarding the environmental stimuli $j$, serving as the predictive variable. The term $\beta$ is the fixed effect of the LME model, which represents the overall association between the biosignal and self-reported stimulus. The term $u_{i}$ represents the random-effect coefficient, while $e_{{\left\{ {ij} \right\}}}$ is the residual. Additionally, a two-sample *t*-test with a significance level of 95% was performed to examine the impact of each type of negative environmental stimuli. The 14 stimuli types presented in the “Data collection” section of this paper were tested. The average value of the nonstimuli and stimuli segment was calculated and compared by each group of negative environmental stimuli in each biosignal. Furthermore, the values were extracted by different lengths of segment, from 5 to 240 s, and repeatedly compared in LME and two-sample *t*-test models to find the length that showed significant differences between the two segments.

### Classification of self-reported environmental stimuli based on biosignals

**Figure 4:**
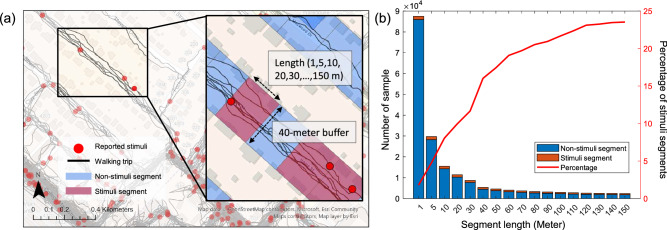
Location-based collective distress: (**a**) overview of line-based segmentation; and (**b**) number of samples by segment length (meter) and presentation of stimuli segments. Images are created using the ArcGIS Pro 3.0, https://www.esri.com/en-us/arcgis/products/arcgis-pro/overview.

We conducted a binary classification to examine location-based collective distress using biosignals collected from daily life (Fig. 4a). Prominent and commonly occurring short-term physical (complexity and variability of gait patterns) and physiological (EDA and BVP) response patterns among individuals exposed to the same location were associated with their emotional and/or physical distress induced by negative environmental stimuli present in the location. Thus, our assumption in capturing location-based collective distress is that there is a distinctive yet common pattern of emotional and/or physical distress that individuals walking in the same location experience in response to negative environmental stimuli.

We first created points of interest (POI) segments using the street centerline from Brazos county with a 40-m buffer. The 40-m buffer width was determined to minimize the overlap with other streets while capturing all relevant POIs from the segment. To test the impact of the POI segment’s length, different lengths were used, such as 1, 5, 10, 20, 30,…, 150 m. Then, geocoded biosignals (black lines) and reported stimuli information (red circles) were added to the map (Fig. 4a). Each set of biosignals (SCL, EDA PSC, stride time, IMU PSC, heart rate, and BVP PSC) was aggregated from all walking trips for each POI segment using the average and standard deviation values, respectively. We also created binary labels using reported stimuli information. If a POI segment contained at least one reported stimulus, it was considered a stimuli segment (red-colored cell). If a POI segment did not contain any reported stimuli information, it was denoted as a nonstimuli segment (blue-colored cell). Figure 4b presents the number of samples in each segment length. For instance, the data from 1-m length contains 1.72% of the minority segment (stimuli segments) and 98.28% of the majority segment (nonstimuli segments).

We used Naïve Bayes to distinguish between the presence and absence of negative environmental stimuli per POI and subject. The Naïve Bayes model was used for its interpretability with a limited amount of data^45^. Additionally, it is fast, yet powerful, and can be utilized to create real-time predictions in actual application scenarios^45^. The performance of the model was assessed with a fivefold cross-validation. It allowed us to capture location-based collective distress patterns of the presence versus absence of negative environmental stimuli. We employed an unweighted average recall (UAR) as a performance matrix because the presence and absence classes were not fully balanced due to the sparse presence of negative environmental stimuli in our real-life study environments^20^.

## Results

### Association results: biosignals and self-reported negative environmental stimuli in real life

We first performed an LME analysis examining the relationship between biosignals and reported stimuli from 67 subjects. Reported stimuli information was considered as the fixed effects. As random effects, we had intercepts for subject identification. The outcomes of the LME analysis are presented in Table 1. The model was significant for stride time (length = 32, beta = 0.33, *p* < 0.05), mean SCL (length = 180, beta = 0.55, *p* < 0.05), SCR amplitude (length = 130, beta = 0.02, *p* < 0.05), SCR frequency (length = 240, beta = 0.60, *p* < 0.05), IMU PSC (length = 20, beta = 0.001, *p* < 0.05), and EDA PSC (length = 46, beta = 0.002, *p* < 0.05). The heart rate and BVP PSC did not show significant results. One possible explanation for these insignificant results is the lagging effect of heart rate–related measures, which has been reported in previous studies^9^. Specifically, IMU and EDA features demonstrate instant and short-term increases and recoveries from stimuli, but heart rate–related features indicate a more gradual pattern of increase and recovery^9^. We assume that this characteristic of heart rate could reduce the performance of statistical tests. Additionally, there is a possibility that heart rate does not show abrupt or detectable changes from the negative affects caused by the built environmental stimuli. On the other hand, PSC measures showed significance in shorter window lengths rather than raw features. One possible reason is that the saliency detection method illuminates fine-grain fluctuations of biosignals in individual walking trip data levels. It calculates the distinctiveness of physiological responses over each segment in contrast to the remaining segments. It helps identify salient data patterns by the negative environmental stimuli, so it may contribute to reducing segment length.

**Table 1:** Linear mixed-effect model results.

Variables/measures	Predictors	Lengths (s)	Beta	*p* value
Raw level	Stride time	32	0.33	< 0.05
	Mean SCL	180	0.55	< 0.05
	SCR amplitude	130	0.02	< 0.05
	SCR frequency	240	0.60	< 0.05
	Mean heart rate	240	− 0.12	0.62
PSC	IMU PSC	20	0.001	< 0.05
	EDA PSC	46	0.002	< 0.05
	BVP PSC	97	0.000	0.16

We further performed a two-sample *t*-test with a significance level of 95% to examine the impact of each type of negative environmental stimuli on biosignals. Table 2 shows that the mean SCL values were significantly higher for the stimuli segments than for the nonstimuli segments of all stimuli types except for Type 3. The stride time was significantly higher for the stimuli segments than for the nonstimuli segments of Types 1–4, 6, 8, 10–11, and 13. Heart rate did not show any significance for all stimuli types. On the other hand, the PSC values were less significant than the raw features in many cases. One possible explanation is that the PSC features were computed by nonfixed-length segments identified in an unsupervised way (bottom-up segmentation) which was described in the “Data preprocessing and feature extraction” section. This method is effective in portraying location fluctuations, but it can reduce the statistical power. For example, if the nonstimuli and stimuli segments were assigned as the same segment in the process of nonfixed-length segmentation, there would be no average difference between the two segments.

**Table 2:** Two-sample *t*-test results.

Type	Negative environmental stimuli	Number of samples	Stride time (s)	Mean SCL (s)	IMU PSC (s)	EDA PSC (s)	BVP PSC (s)
1	Poor walking surface (e.g., cracks, holes)	206	142*	34*	93*	52*	–
2	Blocked sidewalk	62	113*	92 *	–	–	79*
3	Steep sidewalk slope	32	86*	–	77*	–	–
4	Litter (dumping, broken glass, graffiti)	126	137 s*	38 *	–	85*	61*
5	Abandoned vehicle	44	–	98 *	121*	123*	–
6	High speed of traffic	167	91 s*	70 *	–	71*	–
7	Unattended dogs	35	–	86 *	–	–	–
8	Rowdy people	84	105 s*	45 *	–	86*	–
9	Weather condition	68	–	58 *	–	68*	–
10	Lack of sidewalk	212	19*	42 *	–	67*	–
11	Lack of crosswalk	129	158*	58 *	140*	72*	–
12	Lack of benches	46	–	90 *	–	82*	–
13	Lack of crosswalk and pedestrian light	48	60*	75 *	–	139*	–
14	Lack of street trees	13	–	87 *	–	97*	–

*p < 0.05.

### Classification results: location-based collective distress in real life

**Figure 5:**
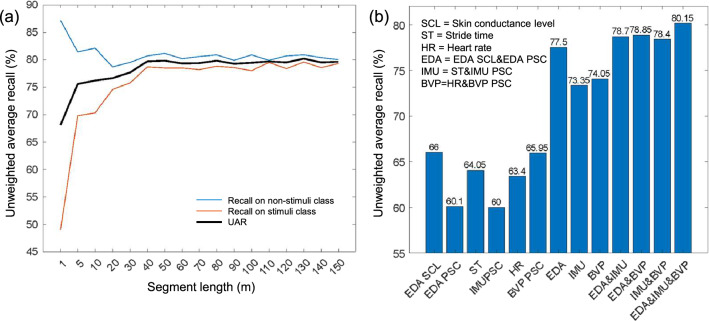
Classification results: (**a**) UAR for segment lengths; and (**b**) UAR for biosignals in 40 m segment length.

We examined the reported negative environmental stimuli in the location-based collective measure using biosignal features. To test the impact of POI lengths, different POI lengths were used in the order of 1, 5, 10, 20, 30,…, 150 m. Figure 5a presents the classification results using UAR on different segment lengths of all biosignal features. The recall of the stimuli and nonstimuli segments are presented in orange and blue, respectively. The black line is the UAR accuracy. We observed that with an increasing segment length, the recall of stimuli class increased, while the recall of nonstimuli class slightly decreased. These evaluation metrics start to saturate to 80% of UAR with a segment length of 40 m, which was selected as the proper segment length for further analysis.

The bar graph in Fig. 5b indicates the performance of features from biosignals using 40 m. Results indicate that EDA-related features performed better compared with IMU and BVP features. Although gait patterns from IMU showed promising results in controlled environments in previous studies^12,30^, the performance in a real-life setting is mitigated. Possible explanations are (1) low IMU data quality due to the waist wear location—a smartphone’s IMU on the waist was used, reducing the sensitivity to smaller gait pattern changes (a single IMU was commonly mounted on the ankle in the controlled environment study); and (2) noise from dynamic and naturalistic experimental conditions—a controlled environment focuses on continuous walking while this real-world study involved many walking intervals/interruptions during a trip as well as confounding behaviors (e.g., checking a cellphone or chatting with other pedestrians).

To better understand the impact of different biosignals on predicting negative environmental stimuli, a machine learning model, Naïve Bayes, was used. The majority of UAR in individual features from the biosignals presented an average of 63.24%. The combined features of raw data and PSC shows an average of 74.97% (+ 11.73% from a single modality). We can observe that if all biosignals are used, the UAR increases up to 80.15% (+ 16.91% from a single modality).

These promising results suggest that the environmental distress of pedestrians can be predicted using the geocoded biosignals collected from real-life settings despite the signal noise and artifacts from uncontrollable confounding factors (e.g., physiology reactivity due to nonintended stimuli and improper sensor placement). This is particularly valuable empirical evidence because (1) the results are from a large data set that covers 191.52 km of walking paths compared with existing research that tends to be limited to a relatively small data size; (2) we demonstrated a new framework for collecting and processing biosignals with the survey data from real-life settings; and (3) to the best of our knowledge, this is the first study to test the feasibility of machine learning algorithms in detecting negative environmental stimuli by correlating biosignals with locations of reported stimuli from participants. Furthermore, results from this study confirm that the existing evidence from observational and lab studies can be expanded to real-life settings. This is the first step toward a better understanding of the relationship between walkability and real-world environmental challenges. These findings have important implications for understanding how biosignals in a real-world setting could be used to detect and reduce the environmental distress of pedestrians.

## Discussion

This study investigated the usefulness of the biosignals collected from free-living individuals in real life to capture the environmental distress they experience as pedestrians. It used large-scale location-based biosignal data acquired from 67 subjects for 2 weeks. Additionally, we used a smartphone-based application to reliably collect and link biosignals and survey data to obtain precise locational information where subjects were distressed during their walking trips. The collected data included 1,561 walking trips with 1,581 locations of stimuli. To the best of our knowledge, this is the largest size of data compared with similar previously published papers. The statistical results showed significant physiological responses to the reported negative environmental stimuli. The spatial analysis with the machine learning model demonstrated UAR up to 80.15% with all modalities of the biosignals. These results provide unique evidence of the usefulness of biosignals from pedestrians in assessing negative environmental stimuli in real life.

The main contribution of this paper is the provision of an accurate estimator to predict the environmental distress of pedestrians by using large-scale biosignals from real-life environments. Most previous research addressing the association between biosignals from real-life settings and built environmental features relies on observations or statistical models. For example, Lee et al.^23,46^ visualized detected biosignal hotspots that were determined from trained models using controlled route data or a certain threshold. Later, they explained what environmental barriers (e.g., side-slope, vertical gap, or unpaved sidewalk) existed around the hotspot locations. Kyriakou et al.^34^ also presented hotspots of pedestrians' biosignals with the locations of their perceived distress, and they drew qualitative results. LaJeunesse et al.^27^ presented a statistical association between biosignals (EDA and BVP) of 21 walking trips from daily life settings and built environmental features (e.g., road type, land use, and so forth). To the best of our knowledge, this is the first study that detects the environmental distress of pedestrians in real-life environments by spatially correlating locations of reported stimuli from participants.

Additionally, we tested the impact of segment length and sensitivity of biosignal features. Although previous research confirmed that apparent physiological reactivity patterns are shown 5–10 s after passing target stimuli in a controlled setting^9^, real-life environments showed the best performance with a 40-m segment length in the prediction model. The results from statistical models also showed significant differences between nonstimuli and stimuli segments from 20 to 240 s. Possible explanations for the wider range of segment/window length of real-life settings compared to controlled route conditions are that (1) longer windows have lower spatial sensitivity compared to shorter windows in biosignals, therefore it may be easier to detect negative stimuli over longer windows in real-life settings; (2) some stimuli types (no sidewalk, steep sidewalk slope, and trees missing) consecutively exist in real environments and can extensively influence physiological reactivity; (3) there would be some deviation between the actual location of the stimuli and the subject-reported location; and (4) complex built environments and diverse traits of pedestrians present uncontrollable confounding variables (e.g., physiology activation due to nonintended stimuli, different perceptions of the same built environmental features, weather, familiarity with the walking paths) that can contaminate the actual effects of various negative environmental stimuli. In terms of the machine learning model, these factors might have mitigated the model’s performance. There have been recent studies on adding confounding variables and contextual information to the machine learning model to increase the model’s performance. Thus, future research can benefit from a more thorough investigation of contextual/confounding factors.

This research also demonstrated a way to collect survey data to establish the labeling set/independent variable. Because research on built environmental assessments in naturalistic ambulatory settings using wearable sensing technologies is still in an exploratory stage, experimental protocols and standards are lacking. For example, a few studies have examined the usefulness of biosignals in real-world data, but the validity of the models has been compromised by the difficulties in demarcating the ground truth for stressful events during walking^23,27,34,46^. They typically used the following approaches to get the ground truth data: (1) qualitative interviews to identify experienced distress events from built environmental features after finishing all biosignal data collection^23,46^; (2) perceptual distress reporting while walking^34^; and (3) field audits by researchers or trained auditors to identify negative environmental stimuli^27^, which are sometimes accompanied by video recording. However, conducting interviews some time after the actual experience is completed can be easily biased. The most accurate way to collect the stimuli location data would be to ask participants to report these locations in real-time while walking. However, this approach will introduce significant signal noises to the data due to the motion and physiological reactivity due to the activity of reporting itself. Last, results from the field audit conducted by another person may not accurately represent the distress level experienced by the subject. For these reasons, this research introduced and tested experimental protocols to collect biosignals, locational information, and personalized ground truth data using a smartphone-based application. Our app-based reporting interface is designed to facilitate accurate reporting of distress experienced during outdoor walking. Thus, subjects can easily and accurately report the locations where participants of the experienced environmental distress. The application is connected with wrist band sensors to collect biosignals and locational information simultaneously. It automatically detects the mode of outdoor trips and displays walking routes immediately after completing each walking trip. To increase the reporting accuracy, participants can enlarge this route map and use the slider bar that also displays the time at a specific location along the traveled walking route. Future research is warranted to examine the associations among biosignals and different ground truth information, including field audits and personalized/collective distress reports by subjects.

In terms of the future application scenario, environmental distress of an urban area will be predicted based on the data available in the specific target area. We expect that the increased prevalence of wearable devices (smartphones and smartwatches) will enable crowdsourcing of a large number of biosignals from citizens, although privacy concerns will need to be addressed carefully. Specifically, smartphone and wristband-type wearable devices are comfortable, inexpensive, widely used, and accurate. Projections indicate that there are 6.65 billion smartphone users worldwide, which means 83.37% of the world’s population owns a smartphone^47^. Regarding smartwatches, roughly one-in-five U.S. adults (21%) say they regularly wear a smartwatch or wearable fitness tracker^48^. Although most of the papers including this study employed specialized sensors (e.g., Empatica E4 wristband) to build a stronger evidence base and draw a widespread consensus pertaining to the usefulness of biosignals in assessing built environmental conditions, it is expected that the data can be collected using off-the-shelf products in the near future. For example, Fitbit, a fitness company, released wristband models (Fitbit Sense) which collect and use EDA, IMU, heart rate, and skin temperature data to track stress levels, although it is not yet possible to export the raw EDA data. Apple watches can measure heart rate, IMU, and ECG data, and the raw data can be downloaded and has been used to detect stress, anxiety, and depression. These indicate the accessibility and availability of these technologies, which will continue to increase.

**Figure 6:**
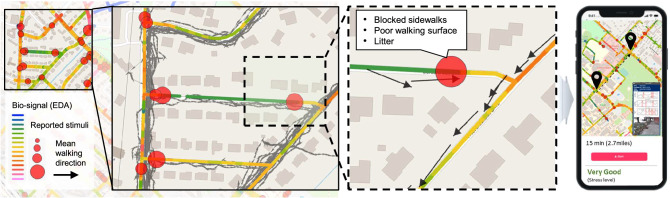
Example of biosignals and reported stimuli visualization and potential application. Images are created using the ArcGIS Pro 3.0, https://www.esri.com/en-us/arcgis/products/arcgis-pro/overview.

Additionally, Fig. 6 presents an example of biosignals and reported stimuli visualization and potential application. Specifically, the aggregated EDA signals of the SCL features along the street segment are presented in a color heat map. The reported stimuli are mapped as a red circle. The size of the circle indicates the normalized frequency of the reported stimuli by the number of walking trips along the segment. Additionally, the black arrow shows the average walking direction for a better understanding of the visual relationship between biosignals and reported stimuli. For example, in the figure with the dotted edge, we can observe that the majority of pedestrians walked from right to left, passing several stimuli (blocked sidewalks, poor walking surfaces, and litter). The color representing normalized SCL by each trip changed from green (SCL between − 0.88 and − 0.64) to yellow (SCL between 0.12 and 0.34); it implies most pedestrians experienced emotional arousal from the stimuli. We envision this information being fed into a navigation and mapping application (e.g., Google Maps, Navmii, or Waze) to show estimated distress levels of the travel routes, including the shortest route and an alternative route with lower distress levels. This approach will offer valuable insights that can be employed in addressing the needs of vulnerable populations (e.g., people with disabilities, older people, pregnant people, and children), thereby helping their mobility planning.

Although the results support the feasibility of our proposed approach in assessing pedestrian distress levels in daily life, there may be several difficulties that limit direct applications in real world practice. Specifically, a smartphone was provided to each participant to collect the phone’s IMU data, and it was installed on the waist to record the gait pattern data. Also, a wristband sensor that was used to capture high-quality biosignals needed for our research. The wearable sensors, however, may cause discomfort to users and have limited accessibility to the general public due to its cost and limited daily utility. The next step of this study is to gather biosignals using a crowdsensing approach. Toward this goal, we plan to develop a platform powered by off-the-shelf wrist sensors and smartphones not requiring any further researcher interventions. After validation, such a platform could be used as an efficient and effective means to identify and remove barriers to creating walkable communities that minimize pedestrian exposure to environmental stressors.

## Conclusion

This work examines the usability of biosignals (electrodermal activity, gait patterns, and heart rate) acquired from real-life settings to capture the environmental distress experienced by pedestrians. We collected location-based large-scale biosignals that cover 191.52 km of walking paths from 67 subjects over 5 months. Our results show that the machine learning algorithm predicted location-based collective distress of pedestrians with 80% accuracy, showing statistical associations between biosignals and the self-reported stimuli. Outcomes from this study is expected to provide new intuition into data-driven built environmental assessment, in which computational systems collaborate with the location-based psychophysiological data to jointly assess spatiotemporal in-the-moment of interest in our urban built environments.

## Data Availability

The datasets generated and/or analyzed during the current study are available from the corresponding author upon reasonable request.

## Abbreviations

BVP: Blood volume pulse
EDA: Electrodermal activity
GPS: Global positioning system
IMU: Inertial measurement unit
LME: Linear mixed-effects
POI: Points of interest
PSC: Physiological saliency cue
SCL: Skin conductance level
SCR: Skin conductance response
UAR: Unweighted average recall
